# Supplementary material for: Disease progression strikingly differs in research and real-world Parkinson’s populations
Source: NPJ Parkinsons Dis. 2024 Mar 13;10:58. doi: 10.1038/s41531-024-00667-5 (PMC10937726; doi:10.1038/s41531-024-00667-5)
Supplement: Supplementary file 1 — Supplementary Materials [file 41531_2024_667_MOESM1_ESM.pdf]

Supplementary Materials

Table of Contents

Supplementary Tables **2**

Supplementary Table 1. Included PD medications. 2

Supplementary Table 2. Real World Data Definition of PD Diagnosis. 3

Supplementary Table 3. Included ICD codes for definition of each Clinical Event. 4

Supplementary Table 4. Regression of Clinical Rating Scale by number of years from PD onset. 10

Supplementary Table 5. Comparison of Hoehn & Yahr progression. 10

Supplementary Table 6. Percentage of Patients with Levodopa Initiation By H&Y Stage (HBS). 11

Supplementary Figures **12**

Supplementary Figure 1. MGB Time from initial PD Diagnosis until last encounter. 12

Supplementary Tables

Supplementary Table 1. Included PD medications.

Carbidopa (Lodosyn)
Artane (Trihexyphenidyl)
Carbidopa / Levodopa Immediate Release (Sinemet)
Benztropine (Cogentin)
Carbidopa / Levodopa Controlled Release (Sinemet CR)
Rotigotine Transdermal Patch (Neupro Patch)
Carbidopa / Levodopa Orally Disintegrating Tablets (Parcopa)
Northera (Droxidopa)
Carbidopa / Levodopa Extended Release Capsules (Rytary or Numient)
Nuplazid (Pimavanserin)
Carbidopa / Levodopa Intestinal Gel (Duopa or Duodopa)
Botox (Botulinum toxin)
Carbidopa / Levodopa and Entacapone (Stalevo)
Dysport (Botulinum toxin)
Entacapone (Comtan)
Myobloc (Botulinum toxin)
Rasagiline (Azilect)
Xeomin (Botulinum toxin)
Deprenyl (Selegiline)
Exelon (Rivastigmine)
Eldepryl (Selegiline)
Aricept (Donepezil)
Zelapar (Selegiline)
Reminyl (Galantamine)
Selegiline Transdermal (Emasm)
Razadyne (Galantamine)
Pramipexole (Mirapex)
Namenda or Namenda XR (Memantine)
Pramipexole Extended Release or Modified Release Tablets (Mirapex ER or Sifrol ER or Pramipexole XR GP)
Ebixa (Memantine)
Adartel (Ropinirole)
Requip (Ropinirole)
Ropinirole Extended Release (Requip XL)
Amantadine (Symmetrel)
Amantadine Extended Release (Gocovri ER or Osmolex ER)
Tolcapone (Tasmar)
Apomorphine (Apokyn)

Parlodel (Bromocriptine)
Safinamide (Xadago or Equfina)
Levodopa / Benserazide Immediate Release (Madopar or Prolopa)
Apo-Trihex (Trihexyphenidyl)
Apomorphine sublingual film (Kynmobi)
Carbidopa / Levodopa and Entacapone Intestinal Gel (Lecigon)
Carbidopa / Levodopa Inhalation Powder (Inbrija)
Levodopa / Benserazide Controlled Release (Madopar CR, Madopar HBS, or Prolopa CR)
Levodopa / Benserazide Dispersible (Madopar Rapid)
Ropinirole [Derived]
Ropinirole Transdermal Patch (Haruropi Tape or HP-3000)
Selegiline [Derived]
Trihexyphenidyl [Derived]
Ethopropazine (Parsitan or Parsidan or Profenamine or Parsidol, or Parkin)
Istradefylline (Nourianz or Nouriast)
Mucuna Pruriens
Opicapone (Ongentys)

Supplementary Table 2. Real World Data Definition of PD Diagnosis.

**Inclusion Codes**	**ICD9**	**ICD10**
Parkinson’s Disease	332, 332.0	G20
**Quiescence exclusion codes**	**ICD9**	**ICD10**
Alzheimer’s Disease & Cerebral Degenerations	331.*	G30*
Dementia	290*	F03.90
Multiple Systems Atrophy/Progressive Supranuclear Palsy	333.0	G90.3, G23.1
Schizophrenia	295*	F20*
Lewy Body Dementia	331.82	G31.83
Encephalitis	323*	G04*
Wilson’s Disease	275.1	E83.01

Supplementary Table 3. Included ICD codes for definition of each Clinical Event.

Diagnosis		Codes
Depression	ICD-9	296.2, 296.20, 296.21, 296.22, 296.23, 296.24, 296.25, 296.26,<br>296.3, 296.30, 296.31, 296.32, 296.33, 296.34, 296.35, 296.36,<br>296.82, 311
	ICD-10	F32.9, F32.0, F32.1, F32.2, F32.3, F32.4, F32.5, F33.40,<br>F33.9, F33.0, F33.1, F33.2, F33.3, F33.40, F33.41, F33.42, F32.89
Fractures	ICD-9	800.00, 800.01, 800.02, 800.03, 800.04, 800.05, 800.06, 800.09, 800.1, 800.11, 800.12,<br>800.13, 800.14, 800.15, 800.16, 800.19, 800.30, 800.31, 800.32, 800.34, 800.35, 800.36,<br>800.39, 800.4, 800.41, 800.42, 800.43, 800.45, 800.46, 800.49, 800.50, 800.51, 800.52,<br>800.53, 800.54, 800.55, 800.56, 800.59, 800.60, 800.61, 800.62, 800.63, 800.64, 800.65,<br>800.66, 800.69, 800.70, 800.71, 800.72, 800.73, 800.74, 800.75, 800.76, 800.79, 800.80,<br>800.81, 800.82, 800.83, 800.84, 800.85, 800.86, 800.89, 800.90, 800.91, 800.92, 800.93,<br>800.94, 800.95, 800.96, 800.99, 801.00, 801.01, 801.03, 801.04, 801.05, 801.06, 801.09,<br>801.10, 801.11, 801.12, 801.13, 801.14, 801.15, 801.16, 801.19, 801.20, 801.21, 801.22,<br>801.23, 801.24, 801.25, 801.26, 801.29, 801.30, 801.31, 801.32, 801.33, 801.34, 801.35,<br>801.36, 801.39, 801.40, 801.41, 801.42, 801.43, 801.44, 801.45, 801.46, 801.49, 801.50,<br>801.51, 801.52, 801.53, 801.54, 801.55, 801.56, 801.59, 801.60, 801.61, 801.62, 801.63,<br>801.64, 801.65, 801.66, 801.69, 801.70, 801.71, 801.72, 801.73, 801.74, 801.75, 801.76,<br>801.79, 801.80, 801.81, 801.82, 801.83, 801.84, 801.85, 801.86, 801.89, 801.90, 801.91,<br>801.92, 801.93, 801.94, 801.95, 801.96, 801.99, 802, 802.0, 802.1, 802.2, 802.20, 802.21,<br>802.22, 802.23, 802.24, 802.25, 802.26, 802.27, 802.28, 802.29, 802.3, 802.30, 802.31,<br>802.32, 802.33, 802.34, 802.35, 802.36, 802.37, 802.38, 802.39, 802.4, 802.5, 802.6,<br>802.7, 802.8, 802.9, 803.00, 803.01, 803.02, 803.04, 803.05, 803.06, 803.09, 803.10,<br>803.11, 803.12, 803.13, 803.14, 803.15, 803.16, 803.19, 803.20, 803.21, 803.22, 803.23,<br>803.24, 803.25, 803.26, 803.29, 803.30, 803.31, 803.32, 803.33, 803.34, 803.35, 803.36,<br>803.39, 803.40, 803.41, 803.42, 803.43, 803.44, 803.45, 803.46, 803.49, 803.50, 803.51,<br>803.52, 803.53, 803.54, 803.55, 803.56, 803.59, 803.60, 803.61, 803.62, 803.63, 803.64,<br>803.65, 803.66, 803.69, 803.70, 803.71, 803.72, 803.73, 803.74, 803.75, 803.76, 803.79,<br>803.80, 803.81, 803.82, 803.83, 803.84, 803.85, 803.86, 803.89, 803.90, 803.91, 803.92,<br>803.93, 803.94, 803.95, 803.96, 803.99, 804.00, 804.01, 804.02, 804.03, 804.04, 804.05,<br>804.06, 804.09, 804.10, 804.11, 804.12, 804.13, 804.14, 804.15, 804.16, 804.19, 804.20,<br>804.21, 804.22, 804.23, 804.24, 804.25, 804.26, 804.29, 804.30, 804.31, 804.32, 804.33,<br>804.34, 804.35, 804.36, 804.39, 804.40, 804.41, 804.42, 804.43, 804.44, 804.45, 804.46,<br>804.49, 804.50, 804.51, 804.52, 804.53, 804.54, 804.55, 804.56, 804.59, 804.60, 804.61,<br>804.62, 804.63, 804.64, 804.65, 804.66, 804.69, 804.70, 804.71, 804.72, 804.73, 804.74,<br>804.75, 804.76, 804.79, 804.80, 804.81, 804.82, 804.83, 804.84, 804.85, 804.86, 804.89,<br>804.90, 804.91, 804.92, 804.93, 804.94, 804.95, 804.96, 804.99, 805.00, 805.01, 805.02,<br>805.03, 805.04, 805.05, 805.06, 805.07, 805.08, 805.10, 805.11, 805.12, 805.13, 805.14,<br>805.15, 805.16, 805.17, 805.18, 805.2, 805.3, 805.4, 805.6, 805.7, 805.8, 805.9, 806,<br>806.00, 806.01, 806.02, 806.03, 806.04, 806.05, 806.06, 806.07, 806.08, 806.09, 806.1,<br>806.10, 806.11, 806.12, 806.13, 806.14, 806.15, 806.16, 806.17, 806.18, 806.19, 806.2,<br>806.20, 806.21, 806.22, 806.23, 806.24, 806.25, 806.26, 806.27, 806.28, 806.29, 806.3,<br>806.30, 806.31, 806.32, 806.33, 806.34, 806.35, 806.36, 806.37, 806.38, 806.39, 806.4,<br>806.5, 806.6, 806.60, 806.61, 806.62, 806.69, 806.7, 806.70, 806.71, 806.72, 806.79,<br>806.8, 806.9, 807, 807.00, 807.01, 807.02, 807.03, 807.04, 807.05, 807.06, 807.07,<br>807.08, 807.09, 807.10, 807.11, 807.12, 807.13, 807.14, 807.15, 807.16, 807.17, 807.18,<br>807.19, 807.2, 807.3, 808, 808.0, 808.1, 808.2, 808.3, 808.4, 808.43, 808.44, 808.49, 809,<br>809.0, 809.1, 810, 810.00, 810.01, 810.02, 810.03, 810.10, 810.11, 810.12, 810.13,<br>811.00, 811.01, 811.02, 811.03, 811.09, 811.10, 811.11, 811.12, 811.13, 811.19, 812,<br>812.00, 812.01, 812.02, 812.03, 812.10, 812.11, 812.12, 812.13, 813, 813.00, 813.01,<br>813.03, 813.04, 813.05, 813.06, 813.07, 813.08, 813.10, 813.11, 813.12, 813.13, 813.14,<br>813.15, 813.16, 813.17, 813.18, 813.20, 813.21, 813.22, 813.23, 813.30, 813.31, 813.32,<br>813.33, 813.40, 813.41, 813.42, 813.43, 813.44, 813.45, 813.46, 813.47, 813.50, 813.51,<br>813.52, 813.53, 813.54, 813.80, 813.81, 813.82, 813.83, 813.90, 813.91, 813.92, 813.93,<br>814.00, 814.01, 814.02, 814.03, 814.04, 814.05, 814.06, 814.07, 814.08, 814.09, 814.10,

		814.11, 814.12, 814.13, 814.14, 814.15, 814.16, 814.17, 814.18, 814.19, 815.00, 815.01, 815.02, 815.03, 815.04, 815.09, 815.10, 815.11, 815.12, 815.13, 815.14, 815.19, 816.00, 816.01, 816.02, 816.03, 816.10, 816.11, 816.12, 816.13, 817.0, 817.1, 818.0, 818.1, 819.0, 819.1, 820.00, 820.01, 820.02, 820.03, 820.09, 820.10, 820.11, 820.12, 820.13, 820.19, 820.20, 820.21, 820.22, 820.30, 820.31, 820.32, 820.8, 820.9, 821.00, 821.01, 821.10, 821.11, 821.20, 821.21, 821.22, 821.23, 821.29, 821.30, 821.31, 821.32, 821.33, 821.39, 822, 822.1, 823.00, 823.01, 823.02, 823.10, 823.11, 823.12, 823.20, 823.21, 823.22, 823.30, 823.31, 823.32, 823.40, 823.41, 823.42, 823.80, 823.81, 823.82, 823.90, 823.91, 823.92, 824.0, 824.1, 824.2, 824.3, 824.4, 824.5, 824.6, 824.7, 824.8, 824.9, 825.0, 825.1, 825.20, 825.21, 825.22, 825.23, 825.24, 825.25, 825.29, 825.3, 825.31, 825.32, 825.33, 825.34, 825.35, 825.39, 826.0, 826.1, 827.0, 827.1, 828.0, 828.1, 829.0, 829.1
	ICD-10	S02.0%%A, S02.0%%A, S06.9%1A, S06.9%2A, S02.0%%A, S06.9%3A, S06.9%4A, S02.0%%A, S06.9%5A, S02.0%%A, S06.9%6A, S06.9%9A, S06.0%9A, S02.0%%A, S06.319A, S06.339A, S06.330A, S02.0%%A, S06.330A, S02.0%%A, S06.331A, S06.332A, S06.313A, S06.314A, S06.323A, S06.324A, S06.333A, S06.334A, S02.0%%A, S06.335A, S02.0%%A, S06.336A, S06.337A, S06.338A, S06.339A, S06.319A, S06.329A, S06.330A, S06.339A, S02.0%%A, S02.0%%A, S06.309A, S06.360A, S02.0%%A, S06.300A, S06.360A, S06.301A, S06.302A, S06.361A, S06.362A, S06.305A, S06.365A, S06.306A, S06.366A, S06.367A, S06.368A, S02.0%%A, S06.309A, S06.369A, S02.0%%A, S06.300A, S06.309A, S06.360A, S02.0%%A, S06.890A, S06.9%0A, S02.0%%A, S06.890A, S06.9%0A, S02.0%%A, S06.891A, S06.892A, S06.9%1A, S06.9%2A, S02.0%%A, S06.893A, S06.894A, S06.9%3A, S06.9%4A, S02.0%%A, S06.896A, S06.897A, S06.898A, S06.9%6A, S06.9%7A, S06.9%8A, S02.0%%A, S06.899A, S06.9%9A, S02.0%%A, S06.899A, S06.9%9A, S02.0%%B, S06.9%1A, S02.0%%B, S02.0%%B, S02.0%%B, S02.0%%B, S06.9%5A, S02.0%%B, S02.101A, S02.102A, S02.109A, S02.110A, S02.111A, S02.112A, S02.113A, S02.118A, S02.119A, S02.11AA, S02.11BA, S02.11CA, S02.11DA, S02.11EA, S02.11FA, S02.11GA, S02.11HA, S02.19%A, S02.101A, S02.102A, S02.109A, S02.101A, S02.102A, S02.109A, S02.101A, S02.102A, S02.109A, S02.101A, S02.102A, S02.109A, S02.101A, S02.102A, S02.109A, S02.101A, S02.102A, S02.109A, S02.101A, S02.102A, S02.109A, S02.101A, S02.102A, S02.109A, S02.101A, S02.102A, S02.109A, S02.102B, S02.109B, S02.110B, S02.111B, S02.112B, S02.113B, S02.118B, S02.119B, S02.11AB, S02.11BB, S02.11CB, S02.11DB, S02.11EB, S02.11FB, S02.11GB, S02.11HB, S02.19%B, S02.101B, S02.101B, S02.102B, S02.109B, S02.101B, S02.102B, S02.109B, S02.101B, S02.102B, S02.109B, S02.101B, S02.102B, S02.109B, S02.101B, S02.102B, S02.109B, S02.2%%A, S02.2%%B, S02.609A, S02.69%A, S02.610A, S02.611A, S02.612A, S02.620A, S02.621A, S02.622A, S02.630A, S02.631A, S02.632A, S02.640A, S02.641A, S02.642A, S02.650A, S02.651A, S02.652A, S02.66%A, S02.670A, S02.671A, S02.672A, S02.600A, S02.601A, S02.602A, S02.609A, S02.69%A, S02.609B, S02.69%B, S02.611B, S02.612B, S02.610B, S02.620B, S02.621B, S02.622B, S02.630B, S02.631B, S02.632B, S02.640B, S02.641B, S02.642B, S02.650B, S02.651B, S02.652B, S02.66%B, S02.670B, S02.671B, S02.672B, S02.600B, S02.601B, S02.602B, S02.609B, S02.69%B, S02.401A, S02.402A, S02.40AA, S02.40BA, S02.40CA, S02.40DA, S02.40EA, S02.40FA, S02.411A, S02.412A, S02.413A, S02.400A, S02.401B, S02.402B, S02.40AB, S02.40BB, S02.40CB, S02.40DB, S02.40EB, S02.40FB, S02.411B, S02.412B, S02.413B, S02.400B, S02.30%A, S02.31%A, S02.32%A, S02.30%B, S02.31%B, S02.32%B, S02.42%A, S02.80%A, S02.81%A, S02.82%A, S02.92%A,

		S02.42%B, S02.80%B, S02.81%B, S02.82%B, S02.92%B, S02.91%A, S02.91%A, S02.91%A, S02.91%A, S02.91%A, S02.91%A, S02.91%B, S02.91%B, S02.91%B, S02.91%B, S02.91%B, S02.91%A, S02.91%A, S02.91%A, S02.92%A, S02.91%A, S02.91%A, S02.91%B, S02.91%B, S02.91%B, S02.91%B, S02.91%B, S12.9%%A, S12.001A, S12.01%A, S12.02%A, S12.030A, S12.031A, S12.040A, S12.041A, S12.090A, S12.091A, S12.000A, S12.101A, S12.110A, S12.111A, S12.112A, S12.120A, S12.121A, S12.130A, S12.131A, S12.14%A, S12.150A, S12.151A, S12.190A, S12.191A, S12.100A, S12.201A, S12.230A, S12.231A, S12.24%A, S12.250A, S12.251A, S12.290A, S12.291A, S12.200A, S12.301A, S12.330A, S12.331A, S12.34%A, S12.350A, S12.351A, S12.390A, S12.391A, S12.300A, S12.401A, S12.430A, S12.431A, S12.44%A, S12.450A, S12.451A, S12.490A, S12.491A, S12.400A, S12.501A, S12.530A, S12.531A, S12.54%A, S12.550A, S12.551A, S12.590A, S12.591A, S12.500A, S12.601A, S12.630A, S12.631A, S12.64%A, S12.650A, S12.651A, S12.690A, S12.691A, S12.600A, S12.9%%A, S12.9%%A, S12.001B, S12.01%B, S12.02%B, S12.030B, S12.031B, S12.040B, S12.041B, S12.090B, S12.091B, S12.000B, S12.101B, S12.110B, S12.111B, S12.112B, S12.120B, S12.121B, S12.130B, S12.131B, S12.14%B, S12.150B, S12.151B, S12.190B, S12.191B, S12.201B, S12.230B, S12.231B, S12.24%B, S12.250B, S12.251B, S12.290B, S12.291B, S12.200B, S12.301B, S12.330B, S12.331B, S12.34%B, S12.350B, S12.351B, S12.390B, S12.391B, S12.300B, S12.401B, S12.430B, S12.431B, S12.44%B, S12.450B, S12.451B, S12.490B, S12.491B, S12.400B, S12.501B, S12.530B, S12.531B, S12.54%B, S12.550B, S12.551B, S12.590B, S12.591B, S12.500B, S12.601B, S12.630B, S12.631B, S12.64%B, S12.650B, S12.651B, S12.690B, S12.691B, S12.600B, S12.9%%A, S22.001A, S22.002A, S22.008A, S22.009A, S22.010A, S22.011A, S22.012A, S22.018A, S22.019A, S22.020A, S22.021A, S22.022A, S22.028A, S22.029A, S22.030A, S22.031A, S22.032A, S22.038A, S22.039A, S22.040A, S22.041A, S22.042A, S22.048A, S22.049A, S22.050A, S22.051A, S22.052A, S22.058A, S22.059A, S22.060A, S22.061A, S22.062A, S22.068A, S22.069A, S22.070A, S22.071A, S22.072A, S22.078A, S22.079A, S22.080A, S22.081A, S22.082A, S22.088A, S22.089A, S22.001B, S22.002B, S22.008B, S22.009B, S22.010B, S22.011B, S22.012B, S22.018B, S22.019B, S22.020B, S22.021B, S22.022B, S22.028B, S22.029B, S22.030B, S22.031B, S22.032B, S22.038B, S22.039B, S22.040B, S22.041B, S22.042B, S22.048B, S22.049B, S22.050B, S22.051B, S22.052B, S22.058B, S22.059B, S22.060B, S22.061B, S22.062B, S22.068B, S22.069B, S22.070B, S22.071B, S22.072B, S22.078B, S22.079B, S22.080B, S22.081B, S22.082B, S22.088B, S22.089B, S22.000B, S32.001A, S32.002A, S32.008A, S32.009A, S32.010A, S32.011A, S32.012A, S32.018A, S32.019A, S32.020A, S32.021A, S32.022A, S32.028A, S32.029A, S32.030A, S32.031A, S32.032A, S32.038A, S32.039A, S32.040A, S32.041A, S32.042A, S32.048A, S32.049A, S32.050A, S32.051A, S32.052A, S32.058A, S32.059A, S32.000A, S32.110A, S32.111A, S32.112A, S32.119A, S32.120A, S32.121A, S32.122A, S32.129A, S32.130A, S32.131A, S32.132A, S32.139A, S32.14%A, S32.15%A, S32.16%A, S32.17%A, S32.19%A, S32.2%%A, S32.10%A, S32.110B, S32.111B, S32.112B, S32.119B, S32.120B, S32.121B, S32.122B, S32.129B, S32.130B, S32.131B, S32.132B, S32.139B, S32.14%B, S32.15%B, S32.16%B, S32.17%B, S32.19%B, S32.2%%B, S32.10%B, S12.9%%A, S22.009A, S32.009A, S32.10%A, S32.2%%A, S22.009B, S32.009B, S32.10%B, S32.2%%B, S12.001A, S12.100A, S12.101A, S12.200A, S12.201A, S12.300A, S12.301A, S12.9%%A, S12.000A, S12.401A, S12.500A, S12.501A, S12.600A, S12.601A, S12.400A, S12.001B, S12.100B, S12.101B, S12.200B, S12.201B, S12.300B, S12.301B, S12.9%%A, S12.000B, S12.401B, S12.500B, S12.501B, S12.600B, S12.601B, S12.400B, S22.019A, S22.029A, S22.039A, S22.049A, S22.059A, S22.009A, S22.069A, S22.079A, S22.089A, S22.009A, S22.019B, S22.029B, S22.039B, S22.049B, S22.059B, S22.009B, S22.069B, S22.079B, S22.089B, S22.009B, S32.019A, S32.029A, S32.039A, S32.049A, S32.059A, S32.009A, S32.2%%A, S32.10%A, S32.2%%B,

		S32.10%B, S22.009A, S12.9%%A, S32.009A, S32.10%A, S12.9%%A, S22.009B, S32.009B, S32.10%B, S22.39%A, S22.31%A, S22.32%A, S22.39%A, S22.42%A, S22.43%A, S22.49%A, S22.41%A, S22.39%B, S22.32%B, S22.39%B, S22.31%B, S22.42%B, S22.43%B, S22.49%B, S22.41%B, S22.21%A, S22.22%A, S22.23%A, S22.24%A, S22.20%A, S22.21%B, S22.22%B, S22.23%B, S22.24%B, S22.20%B, S32.402A, S32.409A, S32.411A, S32.412A, S32.413A, S32.414A, S32.415A, S32.416A, S32.421A, S32.422A, S32.423A, S32.424A, S32.425A, S32.426A, S32.431A, S32.432A, S32.433A, S32.434A, S32.435A, S32.436A, S32.441A, S32.442A, S32.443A, S32.444A, S32.445A, S32.446A, S32.451A, S32.452A, S32.453A, S32.454A, S32.455A, S32.456A, S32.461A, S32.462A, S32.463A, S32.464A, S32.465A, S32.466A, S32.471A, S32.472A, S32.473A, S32.474A, S32.475A, S32.476A, S32.481A, S32.482A, S32.483A, S32.484A, S32.485A, S32.402B, S32.409B, S32.411B, S32.412B, S32.413B, S32.414B, S32.415B, S32.416B, S32.421B, S32.422B, S32.423B, S32.424B, S32.425B, S32.426B, S32.431B, S32.432B, S32.433B, S32.434B, S32.435B, S32.436B, S32.441B, S32.442B, S32.443B, S32.444B, S32.445B, S32.446B, S32.451B, S32.452B, S32.453B, S32.454B, S32.455B, S32.456B, S32.461B, S32.462B, S32.463B, S32.464B, S32.465B, S32.466B, S32.471B, S32.472B, S32.473B, S32.474B, S32.475B, S32.476B, S32.481B, S32.482B, S32.483B, S32.484B, S32.485B, S32.502A, S32.509A, S32.511A, S32.512A, S32.519A, S32.591A, S32.592A, S32.599A, S32.501A, S32.502B, S32.509B, S32.511B, S32.512B, S32.519B, S32.591B, S32.592B, S32.599B, S32.501B, S32.810A, S32.811A, S32.82%A, S32.89%A, S32.9%%A, S22.9%%A, S22.9%%B, S42.002A, S42.009A, S42.001A, S42.012A, S42.013A, S42.014A, S42.015A, S42.016A, S42.017A, S42.018A, S42.019A, S42.022A, S42.023A, S42.024A, S42.025A, S42.026A, S42.021A, S42.032A, S42.033A, S42.034A, S42.035A, S42.036A, S42.031A, S42.002B, S42.009B, S42.001B, S42.012B, S42.013B, S42.014B, S42.015B, S42.016B, S42.017B, S42.018B, S42.019B, S42.022B, S42.023B, S42.024B, S42.025B, S42.026B, S42.032B, S42.033B, S42.034B, S42.035B, S42.036B, S42.031B, S42.101A, S42.102A, S42.109A, S42.122A, S42.123A, S42.124A, S42.125A, S42.126A, S42.121A, S42.131A, S42.132A, S42.133A, S42.134A, S42.135A, S42.136A, S42.142A, S42.143A, S42.144A, S42.145A, S42.146A, S42.151A, S42.152A, S42.153A, S42.154A, S42.155A, S42.156A, S42.112A, S42.113A, S42.114A, S42.115A, S42.116A, S42.191A, S42.192A, S42.199A, S42.101B, S42.102B, S42.109B, S42.122B, S42.123B, S42.124B, S42.125B, S42.126B, S42.121B, S42.132B, S42.133B, S42.134B, S42.135B, S42.136B, S42.142B, S42.143B S42.144B, S42.145B, S42.146B, S42.151B, S42.152B, S42.153B, S42.154B, S42.155B, S42.156B, S42.141B, S42.112B S42.113B, S42.114B, S42.115B, S42.116B, S42.191B, S42.192B, S42.199B, S42.111B, S42.201A, S42.202A, S42.209A S42.212A, S42.213A, S42.214A, S42.215A, S42.216A, S42.221A, S42.222A, S42.223A, S42.224A, S42.225A, S42.226A, S42.231A, S42.232A, S42.239A, S42.241A, S42.242A, S42.249A, S42.211A, S42.291A, S42.292A, S42.293A, S42.294A, S42.295A, S42.296A, S42.252A, S42.253A, S42.254A, S42.255A, S42.256A, S42.202B, S42.209B, S42.201B, S42.212B, S42.213B, S42.214B, S42.215B, S42.216B, S42.221B, S42.222B, S42.223B, S42.224B, S42.225B, S42.226B, S42.231B, S42.232B, S42.239B, S42.241B, S42.242B, S42.249B, S42.211B, S42.292B, S42.293B, S42.294B, S42.295B, S42.296B, S42.291B, S42.252B, S42.253B, S42.254B, S42.255B, S42.256B, S42.251B, S52.002A, S52.009A, S52.101A, S52.102A, S52.109A, S52.90%A, S52.001A, S52.022A, S52.023A, S52.024A, S52.025A, S52.026A, S52.031A, S52.032A, S52.033A, S52.034A, S52.035A, S52.036A, S52.021A, S52.271A, S52.272A, S52.279A, S52.002A, S52.009A, S52.091A, S52.092A, S52.099A, S52.001A, S52.121A, S52.122A, S52.123A, S52.124A, S52.125A, S52.126A, S52.133A, S52.136A, S52109A, S52.189A, S52.009A, S52.109A, S52.90%B, S52.90%C, S52.023B, S52.023C, S52.026B, S52.026C, S52043B, S52.043C, S52.046B, S52.046C, S52.279B, S52.009B, S52.009C, S52.099B, S52.099C,

		S52.123B, S52123C, S52.126B, S52.126C, S52.133B, S52.133C, S52.136B, S52.136C, S52.109B, S52.109C, S52.189B, S52.189C, S52.009B, S52.009C, S52.109B, S52.109C, S52.90%A, S52.309A, S52.209A, S52.209A, S52.309A, S52.90%B, S5290%C, S52.309B, S52.309C, S52.209B, S52.209C, S52.209B, S52.209C, S52.309B, S52.309C, S52.90%A, S52539A, S52.549A, S52.509A, S52.609A, S52.509A, S52.119A, S52.529A, S52.019A, S52.629A, S52.521A, S52.621A, S52.011A, S52.111A, S52.522A, S52.622A, S52.012A, S52.112A, S52.90%B, S52.90%C, S52.539B, S52.539C, S52.509B, S52.509C, S52.609B, S52.609C, S52.509B, S52.509C, S52.609B, S52.609C, S52.90%A, S52.90%A, S52.90%A, S52.90%A, S52.90%B, S52.90%C, S62.109A, S62.009A, S62.123A, S62.126A, S62.113A, S62.116A, S62.163A, S62.166A, S62.173A, S62.176A, S62.183A, S62.186A, S62.133A, S62.136A, S62.143A, S62.146A, S62.153A, S62.156A, S62.109B, 62.009B, S62.123B, S62.126B, S62.113B, S62.116B, S62.163B, S62.166B, S62.173B, S62.176B, S62.183B, S62.186B, S62.133B, S62.136B, S62.143B, S62.146B, S62.153B, S62.156B, S62.309A, S62.233A, S62.236A, S62.319A, S62.349A, S62.329A, 62.359A N, S62.339A, S62.369A, S62.399A, S62.309B, S62.233B, S62.236B, S62.319B, S62.349B, S62.329B, S62.359B, S62.339B, S62.369B, S62.399B, S62.509A, S62.609A, S62.513A, S62.516A, S62.629A, S62.649A, S62.659A, S62.523A, S62.526A, S62.639A, S62.669A, S62.90%A, S62.509B, S62.609B, S62.513B, S62.516B, S62.619B, S62.629B, S62.649B, S62.659B, S62.523B, S62.526B, S62.639B, S62.669B, S62.90%B, S62.90%A, S62.90%B, S62.90%A, S62.90%B, S42.91%A, S52.91%A, S42.92%A, S52.92%A, S22.20%A, S22.49%A, S42.90%A, S52.90%A, S42.91%B, S52.91%B, S42.92%B, S52.92%B, S22.20%B, S22.49%B, S42.90%B, S52.90%B, S72.019A, S72.023A, S72.026A, S72.033A, S72.036A, S72.043A, S72.046A, S72.099A, S72.019B, S72.019C, S72.023B, S72.023C, S72.026B, S72.026C, S72.033B, S72.033C, S72.036B, S72.036C, S72.043B, S72.043C, S72.046B, S72.046C, S72.099B, S72.099C, S72.109A, S72.143A, S72.146A, S72.23%A, S72.26%A, S72.109B, S72.109C, S72.143B, S72.143C, S72.146B, S72.146C, S72.23%B, S72.23%C, S72.26%B, S72.26%C, S72.009A, S72.009B, S72.009C, S72.90%A, S72.309A, S72.90%B, S72.90%C, S72.309B, S72.309C, S72.409A, S72.413A, S72.416A, S72.443A, S72.446A, S72.453A, S72.456A, S72.499A, S72.409B, S72.409C, S72.413B, S72.413C, S72.416, S72.416C, S72.443B, S72.443C, S72.446, S72.446C, S72.453B, S72.453C, S72.456B, S72.456C, S72.499B, S72.499C, S82.009A, S82.009B, S82.009C, S82.109A, S82.839A, S82.101A, S82.831A, S82.102A, S82.832A, S82.109B, S82.109C, S82.839B, S82.839C, S82.101B, S82.831B, S82.102B, S82.832B, S82.209A, S82.409A, S82.201A, S82.401A, S82.202A, S82.402A, S82.209B, S82.209C, S82.409B, S82.409C, S82.201B, S82.401B, S82.202B, S82.169A, S82.819A, S82.161A, S82.811A, S82.311A, S82.821A, 162A Toru, S82.812A, S82.312A, S82.822A, S82.201A, S82.401A, S82.201A, S82.401A, S82.201B, S82.401C, S82.401B, S82.401C, S82.53%A, S82.56%A, S82.53%B, S82.53%C, S82.56%B, S82.56%C, S82.63%A, S82.66%A, S82.63%B, S82.63%C, S82.66%B, S82.66%C, S82.843, S82.843B, S82.843C, S82.846B, S82.846C, S82.853A, S82.856A, S82.846B, S82.846C, S82.899A, S82.899B, S82.899C, S92.009A, S99.009A, S99.019A, S99.029A, S99.039A, S99.049A, S99.099A, S92.009B, S99.009B, S99.019B, S99.029B, S99.039B, S99.049B, S99.099B, S92.819A, S92.909A, S92.109A, S92.253A, S92.256A, S92.213A, S92.223A, S92.226A, S92.309A, S92.201A, S92.202A, S92.209A, S92.819B, S92.109B, S92.253B, S92.256B, S92.213B, S92.216B, S92.223B, S92.226B, S92.309B, S92.201B, S92.202B, S92.209B, S92.403A, S92.406A, S92.503A, S92.506A, S92.403B, S92.406B, S92.503B, S92.506B, S82.90%A, S82.90%B, S72.91%A, S82.91%A, S72.92%A, S82.92%A, S42.90%A, S52.90%A, S72.90%A, S82.90%A, S22.20%A, S22.49%A, S72.90%A, S82.90%A, S72.91%E, S82.91%B, S72.92%E, S82.92%B, S42.90%B, S52.90%B, S72.90%E, S82.90%B, S22.20%B, S22.49%B, S72.90%E, S82.90%B, T14.8%%A, T14.8%%A

Falls	ICD-9	E881.1, E882, E883.0, E883.1, E883.2, E883.9, E884, E884.0, E884.1, E884.2, E884.3, E884.4, E884.5, E884.6, E884.9, E885.9, E886, E886.0, E886.9, E888, E888.0, E888.1, E888.8, E888.9
	ICD-10	W10.0%%A, W10.0%%D, W10.0%%S, W10.1%%A, W10.1%%D, W10.1%%S, W10.2%%A, W10.2%%D, W10.8%%A, W10.8%%D, W10.8%%S, W10.9%%A, W10.9%%D, W11.%%%A, W11.%%%D, W11.%%%S, W12.%%%A, W12.%%%D, W12.%%%S, W13.0%%A, W13.0%%D, W13.1%%A, W13.1%%D, W13.2%%A, W13.2%%D, W13.3%%A, W13.3%%D, W13.4%%A, W13.4%%D, W13.8%%A, W13.8%%D, W13.9%%A, W13.9%%D, W16.011A, W16.011D, W16.012A, W16.012D, W16.021A, W16.021D, W16.022A, W16.022D, W16.031A, W16.031D, W16.032A, W16.032D, W16.111A, W16.111D, W16.112A, W16.112D, W16.121A, W16.121D, W16.122A, W16.122D, W16.131A, W16.131D, W16.132A, W16.132D, W16.211A, W16.211D, W16.212A, W16.212D, W16.221A, W16.221D, W16.222A, W16.222D, W16.311A, W16.311D, W16.312A, W16.312D, W16.321A, W16.321D, W16.322A, W16.322D, W16.331A, W16.331D, W16.332A, W16.332D, W16.41%A, W16.41%D, W16.42%A, W16.42%D, W16.511A, W16.511D, W17.0%%A, W17.0%%D, W17.0%%S, W17.1%%A, W17.1%%D, W17.1%%S, W17.2%%A, W17.2%%D, W17.3%%A, W17.3%%D, W17.4%%A, W17.4%%D, W09.0%%A, W09.0%%D, W09.1%%A, W09.1%%D, W09.2%%A, W09.2%%D, W09.8%%A, W09.8%%D, W15.%%%A, W15.%%%D, W15.%%%S, W07.%%%A, W07.%%%D, W07.%%%S, V00.811A, V00.811D, V00.812A, V00.812D, V00.818A, V00.818D, V00.831A, V00.831D, V00.832A, V00.832D, V00.838A, V00.838D, W05.0%%A, W05.0%%D, W05.1%%A, W05.1%%D, W05.2%%A, W05.2%%D, W06.%%%A, W06.%%%D, W06.%%%S, V00.821A, V00.821D, V00.822A, V00.822D, V00.828A, V00.828D, W08.%%%A, W08.%%%D, W08.%%%S, W18.11%A, W18.11%D, W18.12%A, W18.12%D, V00.891A, V00.891D, V00.892A, V00.892D, V00.898A, V00.898D, W00.1%%A, W00.1%%D, W00.2%%A, W00.2%%D, W14.%%%A, W14.%%%D, W17.81%A, W17.81%D, W17.82%A, W17.82%D, W17.89%A, W17.89%D, V00.181A, V00.181D, V00.182A, V00.182D, V00.211A, V00.211D, V00.212A, V00.212D, V00.221A, V00.221D, V00.222A, V00.222D, V00.281A, V00.281D, V00.282A, V00.282D, V00.381A, V00.381D, V00.382A, V00.382D, W00.0%%A, W00.0%%D, W00.9%%A, W00.9%%D, W01.0%%A, W01.0%%D, W18.2%%A, W18.2%%D, W18.40%A, W18.40%D, W18.41%A, W18.41%D, W18.42%A, W18.42%D, W18.43%A, W18.43%D, W18.49%A, W18.49%D, W03.%%%A, W03.%%%D, V00.188A, V00.188D, V00.218A, V00.218D, V00.228A, V00.228D, V00.288A, V00.288D, V00.388A, V00.388D, W01.10%A, W01.10%D, W01.110A, W01.110D, W01.111A, W01.111D, W01.118A, W01.118D, W01.119A, W01.119D, W18.02%A, W18.02%D, W01.190A, W01.190D, W01.198A, W01.198D, W18.00%A, W18.00%D, W18.01%A, W18.01%D, W18.09%D, W04.%%%A, W04.%%%D, W18.30%A, W18.30%D, W18.31%A, W18.31%D, W18.39%A, W18.39%D, W18.39%S, W19.%%%A, W19.%%%D, W19.%%%S, V00.181A, V00.181D, V00.182A, V00.182D, V00.211A, V00.211D, V00.212A, V00.212D, V00.221A, V00.221D, V00.222A, V00.222D, V00.281A, V00.281D, V00.282A, V00.282D, V00.381A, V00.381D, V00.382A, V00.382D, W00.0%%A, W00.0%%D, W00.9%%A, W00.9%%D, W01.0%%A, W01.0%%D, W18.2%%A, W18.2%%D, W18.40%A, W18.40%D, W18.41%A, W18.41%D, W18.42%A, W18.42%D, W18.43%A, W18.43%D, W18.49%A, W18.49%D

Supplementary Table 4. Regression of Clinical Rating Scale by number of years from PD onset.

	Data Source	Slope	Intercept	r^2 Coefficient	p-value for # years	p-value between groups
**H&Y**	**HBS**	0.05	1.99	0.30	< 0.001	> 0.05
	**MGB**	0.07	2.18	0.24	< 0.001	
**MMSE**	**HBS**	-0.11	28.67	-0.17	< 0.001	< 0.001
	**MGB**	-0.28	25.81	-0.14	<0.001	
**UPDRS Total**	**HBS**	1.54	28.25	0.37	< 0.001	< 0.001
	**MGB**	3.87	18.93	0.29	< 0.001	

Supplementary Table 5. Comparison of Hoehn & Yahr progression.

Hoehn & Yahr Stage	MGB Median Months to Transition to Next Stage	Zhao et al.[1] Median Months to Transition to Next Stage
**1**	18	20
**2**	57	62
**2.5**	29	25
**3**	20	24
**4**	27	26
**5**	NA	NA
**Weighted Expected annual scale increase per year:**	**0.39**	**0.45**

Supplementary Table 6. Percentage of Patients with Levodopa Initiation By H&Y Stage (HBS).

**H&Y Level**	**Levodopa Init**	**Total at HY**	**% Levodopa Init**
**1**	69	136	50.7%
**1.5**	47	74	63.5%
**2**	782	1141	68.5%
**2.5**	373	458	81.4%
**3**	210	261	80.5%
**4**	48	63	76.2%
**5**	19	23	82.6%

Supplementary Figures

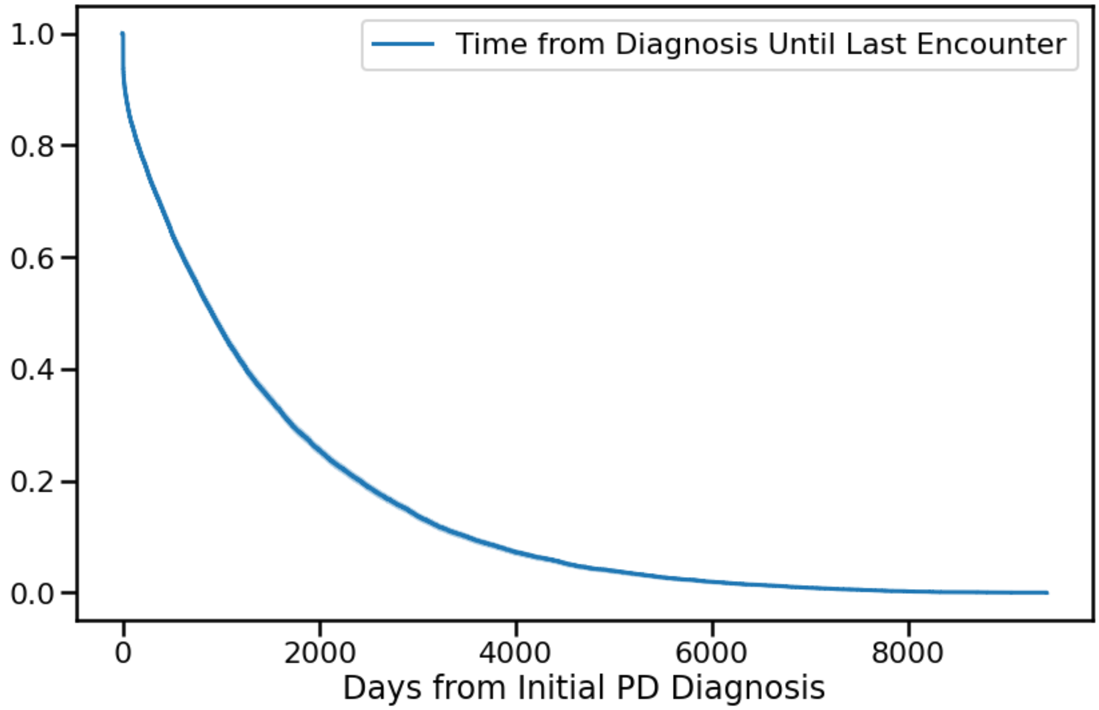

Supplementary Figure 1. MGB Time from initial PD Diagnosis until last encounter.

References

1. Zhao YJ, Wee HL, Chan Y-H, et al. Progression of Parkinson's disease as evaluated by Hoehn and Yahr stage transition times. Mov Disord. 2010;25:710–716.
