# Supplementary material for: Disease progression strikingly differs in research and real-world Parkinson’s populations
Source: NPJ Parkinsons Dis. 2024 Mar 13;10:58. doi: 10.1038/s41531-024-00667-5 (PMC10937726; doi:10.1038/s41531-024-00667-5)
Supplement: Supplementary file 2 — Reporting summary [file 41531_2024_667_MOESM2_ESM.pdf]

Corresponding author(s): Brett Beaulieu-Jones, Clemens Scherzer

Last updated by author(s): Feb 13, 2024

Reporting Summary

Nature Portfolio wishes to improve the reproducibility of the work that we publish. This form provides structure for consistency and transparency in reporting. For further information on Nature Portfolio policies, see our Editorial Policies and the Editorial Policy Checklist.

Statistics

For all statistical analyses, confirm that the following items are present in the figure legend, table legend, main text, or Methods section.

n/a	Confirmed	
☐	☒	The exact sample size (*n*) for each experimental group/condition, given as a discrete number and unit of measurement
☒	☐	A statement on whether measurements were taken from distinct samples or whether the same sample was measured repeatedly
☐	☒	The statistical test(s) used AND whether they are one- or two-sided<br>*Only common tests should be described solely by name; describe more complex techniques in the Methods section.*
☐	☒	A description of all covariates tested
☐	☒	A description of any assumptions or corrections, such as tests of normality and adjustment for multiple comparisons
☐	☒	A full description of the statistical parameters including central tendency (e.g. means) or other basic estimates (e.g. regression coefficient) AND variation (e.g. standard deviation) or associated estimates of uncertainty (e.g. confidence intervals)
☐	☒	For null hypothesis testing, the test statistic (e.g. F, t, r) with confidence intervals, effect sizes, degrees of freedom and P value noted<br>*Give P values as exact values whenever suitable.*
☒	☐	For Bayesian analysis, information on the choice of priors and Markov chain Monte Carlo settings
☒	☐	For hierarchical and complex designs, identification of the appropriate level for tests and full reporting of outcomes
☐	☒	Estimates of effect sizes (e.g. Cohen's *d*, Pearson's *r*), indicating how they were calculated

Our web collection on statistics for biologists contains articles on many of the points above.

Software and code

Policy information about availability of computer code

Data collection	N/a
Data analysis	Extraction code based on prior work: https://github.com/EmilyAlsentzer/PPH-Extract and corresponding mansucript: Alsentzer, Emily, et al. "Zero-shot Interpretable Phenotyping of Postpartum Hemorrhage Using Large Language Models." medRxiv (2023): 2023-05.

For manuscripts utilizing custom algorithms or software that are central to the research but not yet described in published literature, software must be made available to editors and reviewers. We strongly encourage code deposition in a community repository (e.g. GitHub). See the Nature Portfolio guidelines for submitting code & software for further information.

Data

Policy information about availability of data

All manuscripts must include a data availability statement. This statement should provide the following information, where applicable:

- Accession codes, unique identifiers, or web links for publicly available datasets
- A description of any restrictions on data availability
- For clinical datasets or third party data, please ensure that the statement adheres to our policy

Authors had primary access to all data sources used in this study (BKB). All data used in this study are secondary use and therefore the authors must direct those interested in using any data involved to their source:
1. Harvard Biomarkers set is accessible through Accelerating Medicines Partnership program for Parkinson’s disease https://amp-pd.org/unified-cohorts/hbs and

through https://www.bwhparkinsoncenter.org/biobank/
2. Mass General Brigham EMR; https://rc.partners.org/about/who-we-are-risc/research-patient-data-registry
3. Optum® Integrated Claims-EHR Data45 (https://www.optum.com/content/dam/optum3/optum/en/resources/fact-sheets/Integrated-Data-product-sheet.pdf),
4. Fox Insight44 https://foxinsight.michaeljfox.org/

Research involving human participants, their data, or biological material

Policy information about studies with human participants or human data. See also policy information about sex, gender (identity/presentation), and sexual orientation and race, ethnicity and racism.

Reporting on sex and gender	Table 1
Reporting on race, ethnicity, or other socially relevant groupings	NA - not available in two of the source datasets
Population characteristics	Table 1
Recruitment	NA
Ethics oversight	This study was approved by the Mass General Brigham Institutional Review Board (#2017P002452). This study received only de-identified, retrospective data from research datasets (HBS and Fox). Research dataset participants are consented for study and publication as part of the recruitment process. Real world datasets (MGB and Optum) are retrospective de-identified data and therefore individual consent is not required for publication.

Note that full information on the approval of the study protocol must also be provided in the manuscript.

Field-specific reporting

Please select the one below that is the best fit for your research. If you are not sure, read the appropriate sections before making your selection.

☒ Life sciences ☐ Behavioural & social sciences ☐ Ecological, evolutionary & environmental sciences

For a reference copy of the document with all sections, see nature.com/documents/nr-reporting-summary-flat.pdf

Life sciences study design

All studies must disclose on these points even when the disclosure is negative.

Sample size	Table 1 - Retrospective secondary analyses
Data exclusions	Methods - Data Sources and Study population: listed per dataset not copied here due to length
Replication	Comparison between 4 datasets.
Randomization	NA
Blinding	NA

Reporting for specific materials, systems and methods

We require information from authors about some types of materials, experimental systems and methods used in many studies. Here, indicate whether each material, system or method listed is relevant to your study. If you are not sure if a list item applies to your research, read the appropriate section before selecting a response.

Materials & experimental systems

n/a	Involved in the study	
☒	☐	Antibodies
☒	☐	Eukaryotic cell lines
☒	☐	Palaeontology and archaeology
☒	☐	Animals and other organisms
☐	☒	Clinical data
☒	☐	Dual use research of concern
☒	☐	Plants

Methods

n/a	Involved in the study	
☒	☐	ChIP-seq
☒	☐	Flow cytometry
☒	☐	MRI-based neuroimaging

Clinical data

Policy information about clinical studies

All manuscripts should comply with the ICMJE guidelines for publication of clinical research and a completed CONSORT checklist must be included with all submissions.

Clinical trial registration	NA
Study protocol	In Methods - Manuscript
Data collection	Secondary Use
Outcomes	Included in detail in methods

Plants

Seed stocks	*Report on the source of all seed stocks or other plant material used. If applicable, state the seed stock centre and catalogue number. If plant specimens were collected from the field, describe the collection location, date and sampling procedures.*
Novel plant genotypes	*Describe the methods by which all novel plant genotypes were produced. This includes those generated by transgenic approaches, gene editing, chemical/radiation-based mutagenesis and hybridization. For transgenic lines, describe the transformation method, the number of independent lines analyzed and the generation upon which experiments were performed. For gene-edited lines, describe the editor used, the endogenous sequence targeted for editing, the targeting guide RNA sequence (if applicable) and how the editor was applied.*
Authentication	*Describe any authentication procedures for each seed stock used or novel genotype generated. Describe any experiments used to assess the effect of a mutation and, where applicable, how potential secondary effects (e.g. second site T-DNA insertions, mosiacism, off-target gene editing) were examined.*
